# Supplementary material for: Investigating social deprivation and comorbid mental health diagnosis as predictors of treatment access among patients with an opioid use disorder using substance use services: a prospective cohort study
Source: Subst Abuse Treat Prev Policy. 2023 Oct 26;18:59. doi: 10.1186/s13011-023-00568-5 (PMC10605983; doi:10.1186/s13011-023-00568-5)
Supplement: Supplementary file 1 — Supplementary Material 1 [file 13011_2023_568_MOESM1_ESM.docx]

**ADDITIONAL FILE A**

# *Table A: Full negative binominal regression for re-engagement rates with substance use services in the one-year follow-up period*

|  | Unadjusted regression | | Partially adjusted regression ^a^ | | Fully adjusted regression ^b^ | |
| --- | --- | --- | --- | --- | --- | --- |
|  | IRR | 95% CI | IRR | 95% CI | IRR | 95% CI |
| Age | 0.98 | [0.98, 1.00] |  |  | 0.99 | [0.98, 1.00] |
| **Gender** |  |  |  |  |  |  |
| Female | 1 |  |  |  | 1 |  |
| Male | 0.92 | [0.72, 1.20] |  |  | 0.95 | [0.73, 1.24] |
| **Ethnicity** |  |  |  |  |  |  |
| White | 1 |  |  |  | 1 |  |
| Black | 0.74 | [0.47, 1.12] |  |  | 0.71 | [0.44, 1.09] |
| Asian | 1.11 | [0.60, 1.86] |  |  | 1.10 | [0.58, 1.89] |
| Mixed | 1.46 | [0.95, 2.13] |  |  | 1.41 | [0.92, 2.08] |
| Other | 1.29 | [0.68, 2.21] |  |  | 1.41 | [0.72, 2.50] |
| **Marital status** |  |  |  |  |  |  |
| single | 1 |  |  |  | 1 |  |
| married or civil partnership | 1.26 | [0.82, 1.85] |  |  | 1.12 | [0.70, 1.70] |
| divorced, separated, or widowed | 1.25 | [0.83, 1.81] |  |  | 1.46 | [0.95, 2.17] |
| not disclosed/recorded or unknown | 0.81 | [0.59, 1.08] |  |  | 0.81 | [0.55, 1.16] |
| **Social deprivation** |  |  |  |  |  |  |
| Q1 (least deprived) | 1 |  | 1 |  | 1 |  |
| Q2 | 1.17 | [0.79, 1.79] | 1.17 | [0.79, 1.79] | 1.13 | [0.75, 1.76] |
| Q3 (most deprived) | 1.12 | [0.77, 1.69] | 1.12 | [0.77, 1.70] | 1.09 | [0.73, 1.70] |
| no LSOA | 1.12 | [0.72, 1.76] | 1.08 | [0.70, 1.71] | 1.09 | [0.61, 1.98] |
| **Recorded mental health comorbidity** |  |  |  |  |  |  |
| No recorded diagnosis | 1 |  | 1 |  | 1 |  |
| One recorded mental health diagnosis | 1.16 | [0.61, 2.01] | 1.18 | [0.62, 2.05] | 1.00 | [0.49, 1.85] |
| Non-opioid substance use diagnosis | 1.31^*^ | [1.04, 1.67] | 1.32^*^ | [1.04, 1.68] | 1.25 | [0.97, 1.61] |
| Multiple recorded diagnosis | 1.19 | [0.82, 1.68] | 1.18 | [0.81, 1.68] | 1.20 | [0.81, 1.73] |
| **Social fragmentation index** |  |  |  |  |  |  |
| Q1 (least) | 1 |  |  |  | 1 |  |
| Q2 | 1.11 | [0.83, 1.51] |  |  | 1.08 | [0.80, 1.48] |
| Q3 (most) | 0.91 | [0.67, 1.25] |  |  | 0.86 | [0.62, 1.20] |
| no LSOA | 1.00 | [0.71, 1.41] |  |  | ^c^ | ^c^ |
| **Population Density** |  |  |  |  |  |  |
| Q1 (least) | 1 |  |  |  | 1 |  |
| Q2 | 1.13 | [0.84, 1.52] |  |  | 1.11 | [0.82, 1.51] |
| Q3 (most) | 0.99 | [0.72, 1.35] |  |  | 0.93 | [0.67, 1.31] |
| no LSOA | 1.04 | [0.73, 1.46] |  |  | ^c^ | ^c^ |

Exponentiated coefficients; 95% confidence intervals in brackets

^*^ *p* < 0.05, ^**^ *p* < 0.01, ^***^ *p* < 0.001

^a^ Partially adjusted regressions were adjusted for using exposure variables (social deprivation and recorded mental health comorbidity); ^b^ fully adjusted regressions adjusted for exposure and confounders (age, sex, ethnicity, marital status, population density, and social fragmentation); ^c^ colinear with no LSOA group.
