# Supplementary material for: Investigating social deprivation and comorbid mental health diagnosis as predictors of treatment access among patients with an opioid use disorder using substance use services: a prospective cohort study
Source: Subst Abuse Treat Prev Policy. 2023 Oct 26;18:59. doi: 10.1186/s13011-023-00568-5 (PMC10605983; doi:10.1186/s13011-023-00568-5)
Supplement: Supplementary file 2 — Supplementary Material 2 [file 13011_2023_568_MOESM2_ESM.docx]

**ADDITIONAL FILE B**

*Table B: Full negative binominal regressions for contact with crisis and inpatient settings in the one year follow-up period*

|  | Unadjusted regression | | Partially adjusted regression ^a^ | | Fully adjusted regression ^b^ | |
| --- | --- | --- | --- | --- | --- | --- |
|  | IRR | 95% CI | IRR | 95% CI | IRR | 95% CI |
| Age | 0.99 | [0.95,1.03] |  |  | 0.99 | [0.95, 1.02] |
| **Gender** |  |  |  |  |  |  |
| Female | 1 |  |  |  | 1 |  |
| Male | 0.80 | [0.32,1.80] |  |  | 0.73 | [0.32, 1.66] |
| **Ethnicity** |  |  |  |  |  |  |
| White | 1 |  |  |  | 1 |  |
| Black | 1.08 | [0.35,4.47] |  |  | 1.28 | [0.36, 4.90] |
| Asian | 1.52 | [0.31,17.4] |  |  | 1.17 | [0.20, 8.98] |
| Mixed | 0.96 | [0.24,6.19] |  |  | 1.01 | [0.26, 4.63] |
| Other | 0.77 | [0.11,14.1] |  |  | 2.00 | [0.29, 17.9] |
| **Marital status** |  |  |  |  |  |  |
| single | 1 |  |  |  | 1 |  |
| married or civil partnership | 0.82 | [0.22,4.64] |  |  | 1.26 | [0.30, 5.93] |
| divorced, separated, or widowed | 1.07 | [0.31,5.30] |  |  | 1.69 | [0.49, 6.67] |
| not disclosed/recorded or unknown | 0.64 | [0.27,1.68] |  |  | 0.21^**^ | [0.04, 0.90] |
| **Social deprivation** |  |  |  |  |  |  |
| Q1 (least deprived) | 1 |  | 1 |  | 1 |  |
| Q2 | 0.81 | [0.21,2.55] | 0.72 | [0.22,2.17] | 0.85 | [0.24, 2.81] |
| Q3 (most deprived) | 0.44 | [0.12,1.33] | 0.77 | [0.24,2.30] | 0.92 | [0.25, 3.22] |
| no LSOA | 0.80 | [0.19,2.93] | 0.79 | [0.22,2.70] | 0.31 | [0.06, 1.56] |
| **Recorded mental health comorbidity** |  |  |  |  |  |  |
| No recorded diagnosis | 1 |  | 1 |  | 1 |  |
| One recorded mental health diagnosis | 8.95^*^ | [2.27,55.2] | 9.08^**^ | [2.29,56.0] | 8.92^**^ | [1.81, 64.4] |
| Non-opioid substance use diagnosis | 4.46^***^ | [2.15,9.51] | 4.53^***^ | [2.14,9.92] | 3.91^***^ | [1.74, 9.14] |
| Multiple recorded diagnosis | 17.9^***^ | [7.18,50.4] | 17.9^***^ | [6.86,52.3] | 15.9^***^ | [5.89, 47.5] |
| **Social fragmentation index** |  |  |  |  |  |  |
| Q1 (least fragmented) | 1 |  |  |  | 1 |  |
| Q2 | 0.51 | [0.19, 1.33] |  |  | 0.37 | [0.14, 0.99] |
| Q3 (most) | 0.49 | [0.18, 1.28] |  |  | 0.42 | [0.15, 1.13] |
| no LSOA | 0.80 | [0.27, 2.42] |  |  | ^c^ | ^c^ |
| **Population Density** |  |  |  |  |  |  |
| Q1 (least pop) | 1 |  |  |  | 1 |  |
| Q2 | 0.81 | [0.31, 2.08] |  |  | 1.00 | [0.39, 2.59] |
| Q3 (most) | 0.73 | [0.27, 1.94] |  |  | 0.93 | [0.30, 2.82] |
| no LSOA | 1.05 | [0.36, 3.20] |  |  | ^c^ | ^c^ |

Exponentiated coefficients; 95% confidence intervals in brackets

^*^ *p* < 0.05, ^**^ *p* < 0.01, ^***^ *p* < 0.001

^a^ Partially adjusted regressions were adjusted for using exposure variables (social deprivation and recorded mental health comorbidity); ^b^ fully adjusted regressions adjusted for exposure and confounders (age, sex, ethnicity, marital status, population density, and social fragmentation); ^c^ colinear with no LSOA group.
