# Supplementary material for: Investigating social deprivation and comorbid mental health diagnosis as predictors of treatment access among patients with an opioid use disorder using substance use services: a prospective cohort study
Source: Subst Abuse Treat Prev Policy. 2023 Oct 26;18:59. doi: 10.1186/s13011-023-00568-5 (PMC10605983; doi:10.1186/s13011-023-00568-5)
Supplement: Supplementary file 3 — Supplementary Material 3 [file 13011_2023_568_MOESM3_ESM.docx]

**ADDITIONAL FILE C**

*Table C: Cox regressions for contact with crisis and inpatient settings in the one year follow-up and tests of proportional hazards assumption*

|  | Unadjusted regression | | Partially adjusted regression ^a^ | | Fully adjusted regression ^b^ | |
| --- | --- | --- | --- | --- | --- | --- |
|  | HR | 95% CI | HRR | 95% CI | HR | 95% CI |
| Age | 0.99 | [0.97,1.02] |  |  | 0.98 | [0.95,1.02] |
| **Gender** |  |  |  |  |  |  |
| Female | 1 |  |  |  | 1 |  |
| Male | 0.98 | [0.53,1.79] |  |  | 1.05 | [0.55,2.01] |
| **Ethnicity** |  |  |  |  |  |  |
| White | 1 |  |  |  | 1 |  |
| Black | 1.36 | [0.61,3.04] |  |  | 0.92 | [0.39,2.17] |
| Asian | 1.91 | [0.68,3.04] |  |  | 1.35 | [0.43,4.22] |
| Mixed | 1.20 | [0.43,3.35] |  |  | 0.90 | [0.31,2.60] |
| Other | 1.91 | [0.59,6.20] |  |  | 1.50 | [0.37-6.00] |
| **Marital status** |  |  |  |  |  |  |
| single | 1 |  |  |  | 1 |  |
| Married or civil partnership | 1.22 | [0.48,3.08] |  |  | 1.06 | [0.37,2.99] |
| divorced, separated, or widowed | 1.45 | [0.62,3.41] |  |  | 2.55 | [0.98,6.66] |
| not disclosed/recorded or unknown | 0.75 | [0.37,1.49] |  |  | 0.36 | [0.11,1.21] |
| **Social deprivation** |  |  |  |  |  |  |
| Q1 (least deprived) | 1 |  | 1 |  | 1 |  |
| Q2 | 0.88 | [0.39,1.98] | 0.88 | [0.39,1.98] | 0.77 | [0.33,1.79] |
| Q3(most deprived) | 0.67 | [0.30,1.50] | 0.67 | [0.30,1.50] | 0.76 | [0.32,1.81] |
| no LSOA | 0.84 | [0.34,2.06] | 0.84 | [0.34,2.06] | 0.43 | [0.12,1.57] |
| **Recorded mental health comorbidity** |  |  |  |  |  |  |
| No recorded diagnosis | 1 |  | 1 |  | 1 |  |
| One recorded mental health diagnosis | 9.62^***^ | [3.78,24.44] | 9.62^***^ | [3.78,24.44] | 7.77^***^ | [2.73,22.08] |
| Non-opioid substance use diagnosis | 2.60^**^ | [1.31,5.15] | 2.60^**^ | [1.31,5.15] | 2.30^*^ | [1.12,4.73] |
| Multiple recorded diagnosis | 7.18^***^ | [3.46,14.91] | 7.18^***^ | [3.46,14.91] | 7.05^***^ | [3.19,15.56] |
| **Social fragmentation index** |  |  |  |  |  |  |
| Q1 (least fragmented) | 1 |  |  |  | 1 |  |
| Q2 | 1.03 | [0.52,2.05] |  |  | 1.08 | [0.53,2.22] |
| Q3 | 0.96 | [0.48,1.92] |  |  | 0.80 | [0.38,1.68] |
| no LSOA | 1.07 | [0.49,2.31] |  |  | ^c^ | ^c^ |
| **Population Density** |  |  |  |  |  |  |
| Q1 (least pop) | 1 |  |  |  | 1 |  |
| Q2 | 1.16 | [0.62,2.21] |  |  | 1.17 | 1.16 |
| Q3 | 0.69 | [0.33,1.46] |  |  | 0.65 | 0.69 |
| no LSOA | 1.03 | [0.49,2.17] |  |  | ^c^ | ^c^ |

Exponentiated coefficients; 95% confidence intervals in brackets

^*^ *p* < 0.05, ^**^ *p* < 0.01, ^***^ *p* < 0.001

^a^ Partially adjusted regressions were adjusted for using exposure variables (social deprivation and recorded mental health comorbidity); ^b^ fully adjusted regressions adjusted for exposure and confounders (age, sex, ethnicity, marital status, population density, and social fragmentation); ^c^ colinear with no LSOA group.
